# Targeted capture sequencing identifies genetic variations of *GRK4* and *RDH8* in Han Chinese with essential hypertension in Xinjiang

**DOI:** 10.1371/journal.pone.0255311

**Published:** 2021-07-23

**Authors:** Wenxi Jiang, Xizi Wang, Ronghui Li, Panpan Wang, Guangle Shan, Xiaodong Jia, Mingliang Gu

**Affiliations:** 1 Department of Medicine, The Fifth Affiliated Hospital of Xinjiang Medical University, Urumqi, China; 2 Joint Laboratory for Translational Medicine Research, Beijing Institute of Genomics, Chinese Academy of Sciences & Liaocheng People’s Hospital, Liaocheng, China; 3 CAS Key Laboratory of Genome Science and Information, Beijing Institute of Genomics, Chinese Academy of Sciences (CAS), Beijing, China; George Washington University School of Medicine and Health Sciences, UNITED STATES

## Abstract

Essential hypertension is a common cardiovascular disease with complex etiology, closely related to genetic and environmental factors. The pathogenesis of hypertension involves alteration in vascular resistance caused by sympathetic nervous system (SNS) and renin angiotensin system (RAS). Susceptibility factors of hypertension vary with regions and ethnicities. In this study, we conducted target capture sequencing on 54 genes related to SNS and RAS derived from a collection of Han nationality, consisting of 151 hypertension patients and 65 normal subjects in Xinjiang, China. Six non-synonymous mutations related to hypertension were identified, including *GRK4* rs1644731 and *RDH8* rs1801058, Mutations are predicted to affect 3D conformation, force field, transmembrane domain and RNA secondary structure of corresponding genes. Based on protein interaction network and pathway enrichment, *GRK4* is predicted to participate in hypertension by acting on dopaminergic synapse, together with interacting components. *RDH8* is involved in vitamin A (retinol) metabolism and consequent biological processes related to hypertension. Thus, *GRK4* and *RDH8* may serve as susceptibility genes for hypertension. This finding provides new genetic evidence for elucidating risk factors of hypertension in Han nationality in Xinjiang, which in turn, enriches genetic resource bank of hypertension susceptibility genes.

## Introduction

Essential hypertension (EH) is traditionally defined as persistently high blood pressure (HBP) with systolic blood pressure ≥ 140 mmHg and diastolic blood pressure ≥ 90 mmHg. The prevalence of hypertension is expected to increase by 60% worldwide by 2025 [1]. High blood pressure is one of the leading causes of death in China. However, awareness, treatment and control rate of hypertension in China remains at a relatively low level (46.9%, 40.7% and 15.3%, respectively) [2–4].

Many pathogenic elements contribute to EH, including smoking, drinking, high salt, oil intake, air pollution [5] and genetic alterations. Genetic and environmental factors can affect intermediate phenotypes [6], including SNS and RAS, sodium excretion, vascular reactivity and cardiac contraction. Generally, SNS is the first defense against a wide range of environmental pressures, and neuro-modulatory factors respond very rapidly during blood pressure regulation. Among various genetic risk factors, over-activation of SNS can cause both hypertension and target organ damage [7, 8]. The main functions of RAS are to regulate blood pressure and maintain the homeostasis of water and electrolyte, which is an important mechanism involved in the pathogenesis of hypertension. Renin hydrolyzes angiotensinogen in the plasma to produce angiotensinogen I (Ang I). Ang I exerts a weak effect on vasoconstriction, however, when entering pulmonary circulation, it is converted into Ang II catalyzed by angiotensin-converting enzyme (ACE). As a very strong vasoactive substance, Ang II increases susceptibility to hypertension [9].

The development and progression of EH can be affected by different genetic factors in different populations and in different regions. At present, very few studies have been focused on genetic factors related to hypertension in Xinjiang Han nationality. The next-generation sequencing enables to explore pathogenic variation within whole genome or exome. For hypertension, a common disease with high genetic heterogeneity, targeted sequencing may greatly improve detection efficiency by directly correlating candidates with SNS or RAS [10, 11]. In this study, we aim to identify genetic factors of hypertension in Xinjiang Han population by targeted capture sequencing on 54 genes associated with SNS or RAS.

## Materials and methods

### Research objects

According to diagnostic criteria (Chinese Guidelines for Prevention and Treatment of Hypertension, 2009 Basic Version), in the absence of antihypertensive drugs, hypertension was diagnosed as systolic blood pressure ≥140 mm Hg and/or diastolic blood pressure ≥90 mm Hg measured three times on the same day. The patients with a history of hypertension and currently taking antihypertensive drugs were diagnosed as hypertension even with blood pressure lower than 140/90mm Hg. From March 2015 to December 2015, 151 patients with hypertension treated at the Fifth Affiliated Hospital of Xinjiang Medical University were randomly selected as “case” group, and 65 healthy adults without hypertension, metabolic diseases or family history of hypertension served as “control” group. All subjects were Han ethnicity, born in Xinjiang. This study was approved by Ethics Committee of the Fifth Affiliated Hospital of Xinjiang Medical University. Informed consent was signed by each subject before blood sample collection.

### Selection of candidate genes

Both SNS and RAS regulate blood pressure through a variety of physiological and cellular pathways. We selected 54 coding genes involved in SNS or RAS for targeted sequencing, and adopted systematic analysis to deeply mine genetic factors related to hypertension (S1 Table).

### Targeted capture and next-generation sequencing

Genomic DNA was extracted from peripheral blood samples using QIAamp DNA blood maxi kit. Then DNA was interrupted into ~200 bp fragments with Covaris S220. Breaking parameters were setup as follows: Duty factor 10%; Peak Incident Power 175; Cycles per Burst 200; Treatment time 360s; and Bath Temperature 4°C -8°C. Agilent 2100 quality control was performed on fragmented DNA. Agilent Sureselect DNA targeting sequence capture kit was used for library preparation (S2 Table). Firstly, fragmented DNA was end-repaired and purified using AMPure XP beads. The purified DNA was added with A at the 3 ’end and purified with AMPure XP beads. Subsequently, DNA was connected with an adaptor and purified with AMPure XP beads. Secondly, polymerase chain reaction (PCR) was performed to expand the linker. Library preparation was programed as follows: pre-denaturation at 94°C for 2 min; denaturation at 94°C for 30 s, annealing at 65°C for 40 s, extension at 72°C for 2 min, a total of 30 cycles of the above steps; extension at 72°C for 10 min; with a total amplification volume of 50 μl. Agilent 2100 was used for quality control. Finally, Illumina Nextseq500CN was applied for sequencing analysis. Using Trimmatomatic [12] to remove the original sequencing connector and low-quality sequence. Filtered sequence was aligned to Thousands Genome Reference Sequence (GRCh37) using BWA [13]. The output data were converted to BAM file and sorted with Samtools [14]. Sequences were deduplicated with Picard. Using GATK software [15], single nucleotide variation (SNV) and indel mutation (In/Del) were analyzed and filtered (S1 File). The called variants were mapped to dbSNP database and annotated with ANNOVAR [16].

### Quality control (QC) and Allelic association

To obtain high-quality data for association analysis, SNPs were trimmed using the following criteria: (1) call rate of sample or SNP > 95%; (2) a threshold of 0.0001 for Hardy-Weinberg equilibrium (HWE). Generally, SNPs with minor allele frequencies (MAFs) > 0.01 were included in association analysis. Sequencing depth was counted by R program. Samples with sequencing depth less than 30 were removed. Using QC-passed SNPs to calculate allele frequency difference and allele superiority ratio between case and control, SNPs related to EH were included (*P*<0.05). QC and Allelic association were completed through Plink [17].

### Protein 3D structure

Swiss-model was used to construct 3D configuration of *GRK4* and *RDH8* encoded proteins based on amino acid sequences. Then, SwissPDB viewer was used to analyze structural changes before and after mutation was introduced and to estimate alterations in force fields.

### RNA secondary structure

RNA-fold sever and RNAstructure were used to predict RNA secondary structure of *GRK4* and *RDH8*. The minimum free energy prediction model was applied. Dynamic changes were observed before and after the minimum free energy mutation was introduced.

### PPI network construction and function enrichment analysis

STRING [18], IntAct [19], MINT [20], BioGRID [21], HTRIdb [22], InWeb_IM [23] and HPRD [24] were employed to query interaction proteins of GRK4 and RDH8. Protein-protein interaction (PPI) network was constructed by using Cytoscape. Functional enrichment was established with David (https://david.ncifcrf.gov/).

## Results

### SNP distribution

**Fig 1:**
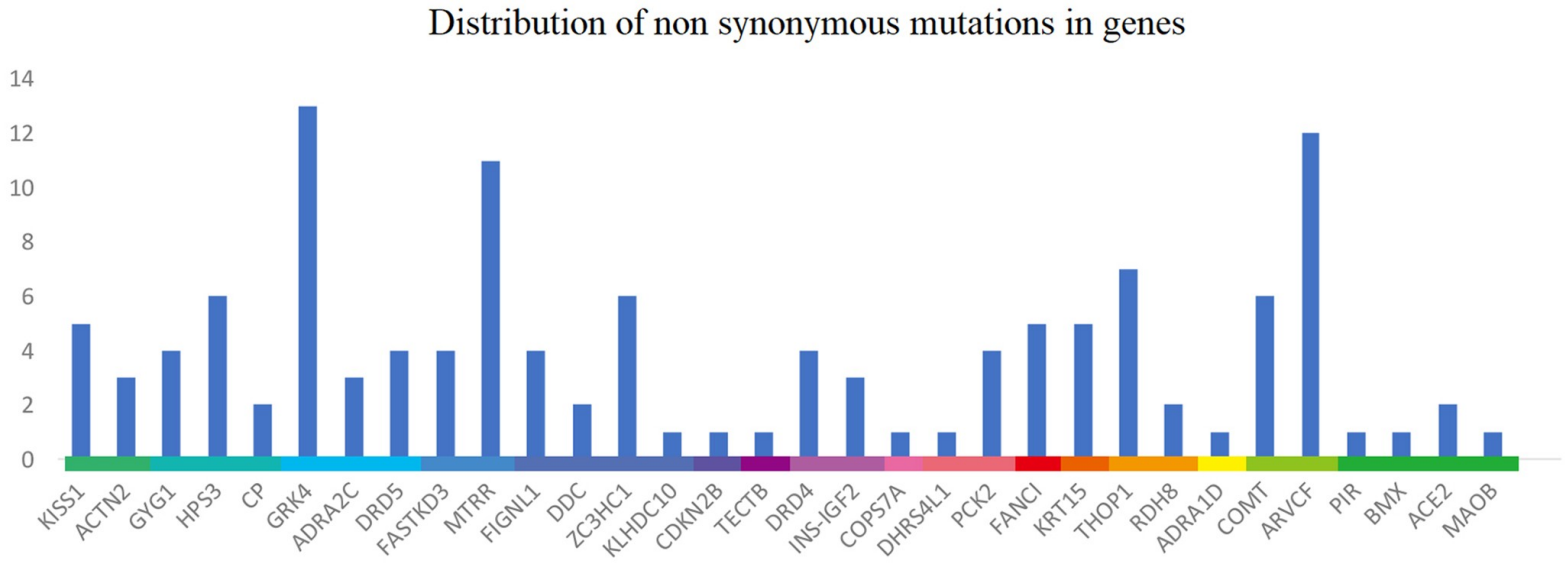
Distribution of non-synonymous mutations. The horizontal, coordinate and vertical axis represents genes, chromosomes, and SNP number, respectively.

A total of 17,581 SNPs was obtained after comparing sequencing results with dbSNP database. These SNPs were annotated using ANNOVAR. Most SNPs were located in intergenic regions and introns. 213 SNPs were localized in exons, including 137 non-synonymous mutations within 32 targeted sequencing genes (Fig 1). Because of the presence of 2 kb extension flanking each sequence, some SNPs were located beyond targeted capture sequencing genes.

Through quality control parameter screening, 148 samples met enrollment criteria, including 109 cases and 39 controls carrying 8,761 eligible SNPs. Based on plink, 451 SNPs were related to hypertension (*p* <0.05). Through annotation, 6 SNPs (non-synonymous mutations) were located in exons (Table 1). The protein encoded by *HPS3* may participate in organelle biogenesis associated with melanosomes, platelet dense granules and lysosomes. Related diseases include Hermansky Pudlak syndrome 3 and Hermansky Pudlak syndrome without pulmonary fibrosis. A family member of Fas activated serine/threonine kinase domain (FASTKD) encoded by *FASTKD3* may be involved in apoptosis. The protein encoded by *FASTKD3* interacts with certain components of mitochondrial respiration and translation. *FASTKD3* is associated with Sengers syndrome. *FIGNL1* encodes a protein belonging to AAA ATPase family. *FIGNL1* is absorbed into DNA damage site and participates in DNA double strand break repair through homologous recombination. In addition, FIGNL1 can be located in centrosome, inhibit cilia formation, and regulate the proliferation and differentiation of osteoblasts. *HPS3*, *FASTKD3* and *FIGNL1* 1 are not directly or indirectly associated with hypertension. The mutation frequencies of these three genes as well as *GRK4* rs77833898 were low, with OR value of less than 1. Therefore, in this study, we focused on how *RDH8* rs1644731 and *GRK4* rs1801058 correlate with hypertension in Han people in Xinjiang. The functions of HPS3, FASTKD3 and FIGNL1 have no direct or indirect association with the pathogenesis of hypertension, and the existing studies have not proved their association with hypertension. The frequency of mutations in these three loci and GRK4 rs77833898 was low in the samples, and the OR values were all less than 1. Therefore, this study mainly studied how the two genes, RDH8 rs1644731 and GRK4 rs1801058, and their mutation sites play a role in the pathogenesis of hypertension in Han people in Xinjiang.

**Table 1:** SNPs significantly associated with hypertension.

Gene	SNP	Allele	AFF	UNAFF	*P*	OR	95% CI
RDH8	rs1644731	T/C	114/104	24/54	0.001074	2.466	2.3097–2.5413
RDH8	rs77833898	G/A	3/215	7/71	0.001435	0.1415	0.1189–0.1681
HPS3	rs142027515	C/T	0/218	2/76	0.01768	0	-0.0093–0.0093
FASTKD3	rs3733782	T/A	1/217	3/75	0.02617	0.1152	0.1021–0.1284
GRK4	rs1801058	T/C	114/104	30/48	0.03595	1.754	1.6750–1.8339
FIGNL1	rs10235371	C/T	4/214	5/73	0.04341	0.2729	0.2537–0.2922

G protein-coupled receptor kinase 4 (*GRK4*), located on chromosome 4, has 23 exons. The rs1801058 is located in exon 20. GRK4 regulates blood pressure by modifying dopamine signal in kidney [25]. A high level of phosphorylated dopamine D1 receptor (D1R) contributes to HBP. Notably, activities of dopamine receptor and AT1R are regulated by GRK4 and protein phosphatase mediated phosphorylation/dephosphorylation [9, 26]. All-trans retinol dehydrogenase coding gene (*RDH8*), also known as photoreceptor RDH, located on chromosome 19, has 6 exons. The rs1644731 is located in exon 5. Alcohol reduction is the first step of rhodopsin regeneration pathway [27, 28].

### Protein 3D structure analysis

**Fig 2:**
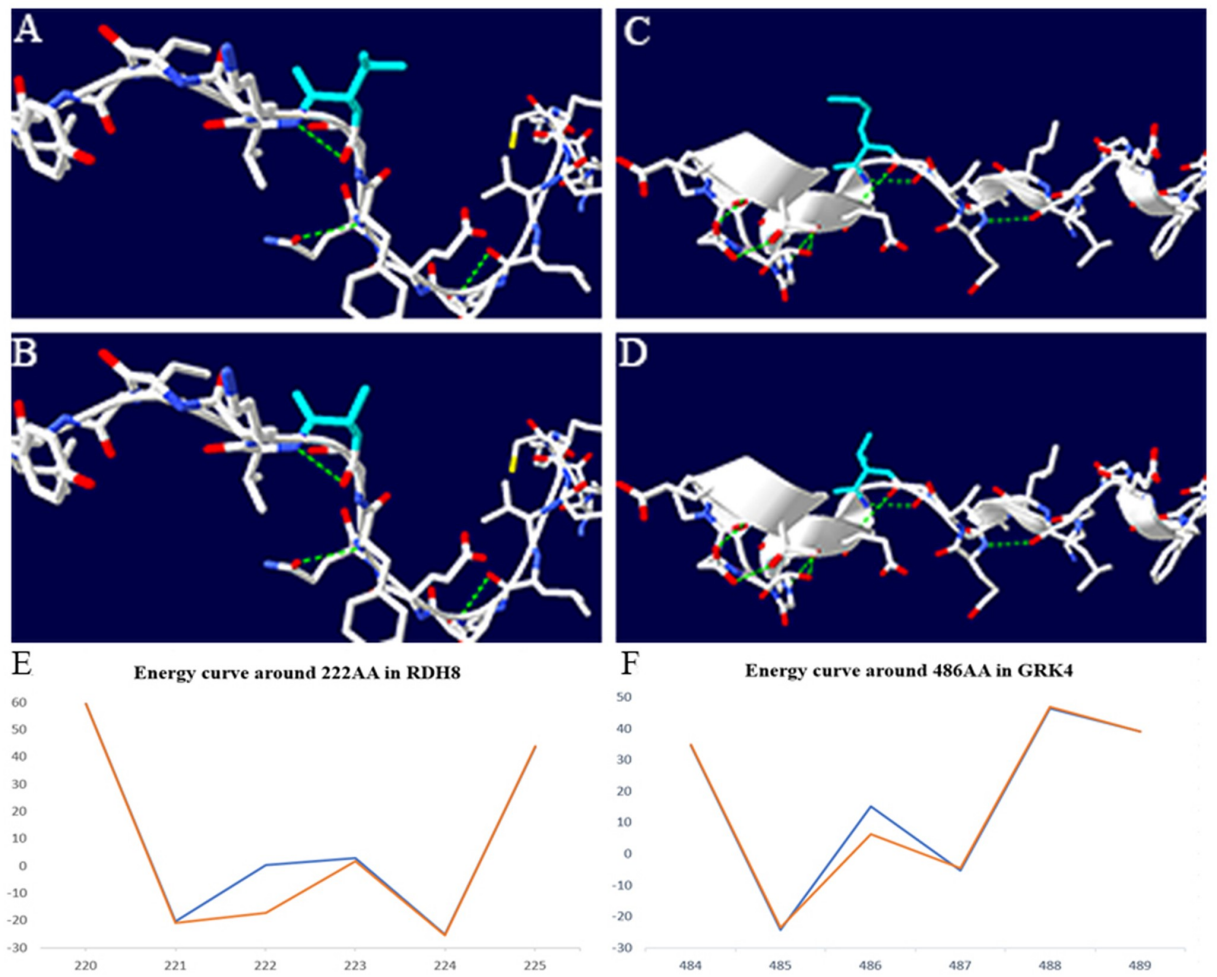
Schematic diagram of 3D protein structure and force field curves. A, B light blue represents amino acid *486 Val / Ala* before and after mutation in *GRK4*; C, D light blue represents amino acid *222 Met / Thr* before and after mutation in *RDH8*. The green dotted line represents hydrogen bonding. E. The blue line represents *RDH8 p*. *222M*. The red line represents *RDH8 p*. *222T*; F. The blue line represents *GRK4 p*. *486V*. The red line represents *GRK4 p*. *486A*.

Mutations in coding regions may alter protein structure, and then affect protein function. Mutations in rs1644731 and rs1801058 caused amino acid *486* in *GRK4* to change from valine (V) to alanine (A), whereas amino acid *222* in *RDH8* from methionine (M) to serine (T), respectively. In order to decipher changes in configuration, tertiary structure of *GRK4* or *RDH8* was constructed by Swiss-model, whereas 3D structure by SwissPDB-viewer (Fig 2).

Potential energy changes in *GRK4* (486 V →A) and *RDH8* (222 M→T) were estimated by GROMOS96 field using SwissPDB-viewer (Fig 2). Mutations are predicted to cause instability of protein structure.

### Analysis of transmembrane structure domain

TMpred was used to predict transmembrane domains of protein sequences upon *GRK4 p*.*V486A* and *RDH8 p*.*M222T*. One transmembrane domain model was predicted in *GRK4*, including one transmembrane helix. No significant change in transmembrane domain was predicted in response to individual mutation. Two models of transmembrane domains were predicted in *RDH8*, including three and two transmembrane helices, respectively. The amino acid as start- or end-position of transmembrane helix changed when sequence mutation was introduced (Table 2). Based on UniProt annotation, *RDH8* is a multi-channel transmembrane protein. Amino acid changes may affect RDH8 transmembrane transport.

**Table 2:** Distribution of transmembrane domains before and after *GRK4* and *RKH8* site mutations.

Gene	Model	From	To	Length	Score	Orientation
GRK4 p.486V/A	1	363	383	21	503	i-o
RDH8 p. 222M	1	3	23	21	685	i-o
		135	158	24	848	o-i
		165	183	19	901	i-o
	2	8	26	19	851	o-i
		136	155	20	1111	i-o
RDH8 p. 222T	1	23	43	21	666	i-o
		155	178	24	848	o-i
		185	203	19	901	i-o
	2	28	46	19	851	o-i
		156	175	20	1111	i-o

Model: model of predicted transmembrane domain; From: start position of transmembrane helix; To end position of transmembrane helix; Length: length of transmembrane helix; i: intracellular membrane; o: extracellular membrane

### RNA secondary structure prediction analysis

Using RNAstructure, secondary structure of *GRK4* or *RDH8* mRNA was constructed (S1 Fig). The minimum free energy of *GRK4 m*.*1457T* or *m*.*1457C* was -643.6 Kcal/mol or -645.1 Kcal/mol, respectively. The minimum free energy of *RDH8 m*.*665T* or *m*.*665C* was -779.0 Kcal/mol or -779.7 Kcal/mol, respectively. Mutations in *GRK4* m.1457 and *RDH8* m.665 did not change free energy of RNA secondary structure, which might have little effect on mRNA stability.

### PPI network analysis for *GRK4* and *RDH8*

**Fig 3:**
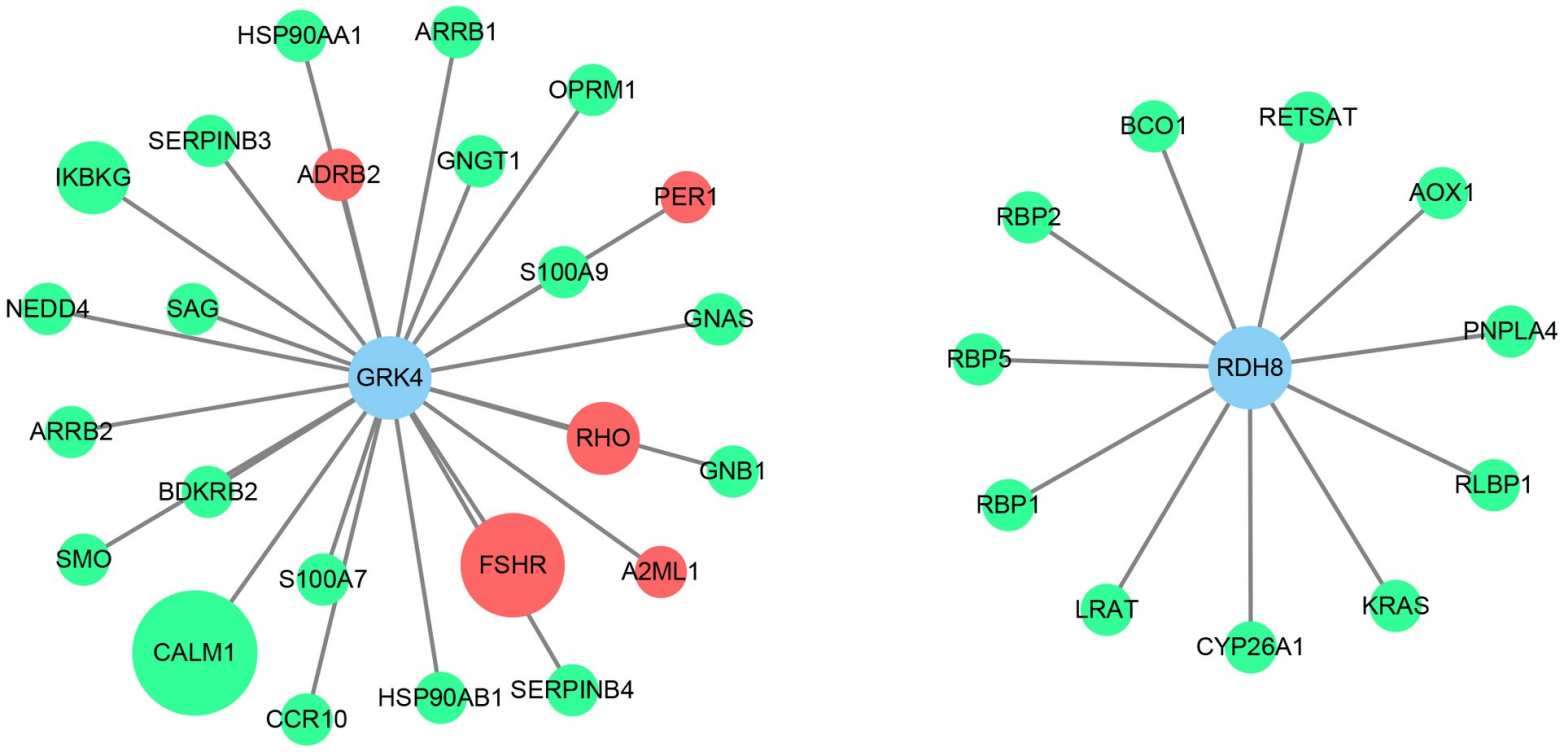
Protein-protein interaction network. Red: genes have been reported to be related to hypertension. No evidence has been provided to correlate green genes with hypertension. The larger the circle, the more databases query this interaction relationship (*CALM1* was queried in five databases; *FSHR* in four; *RHO* and *IKBKG* in two; whereas others in one database).

**Fig 4:**
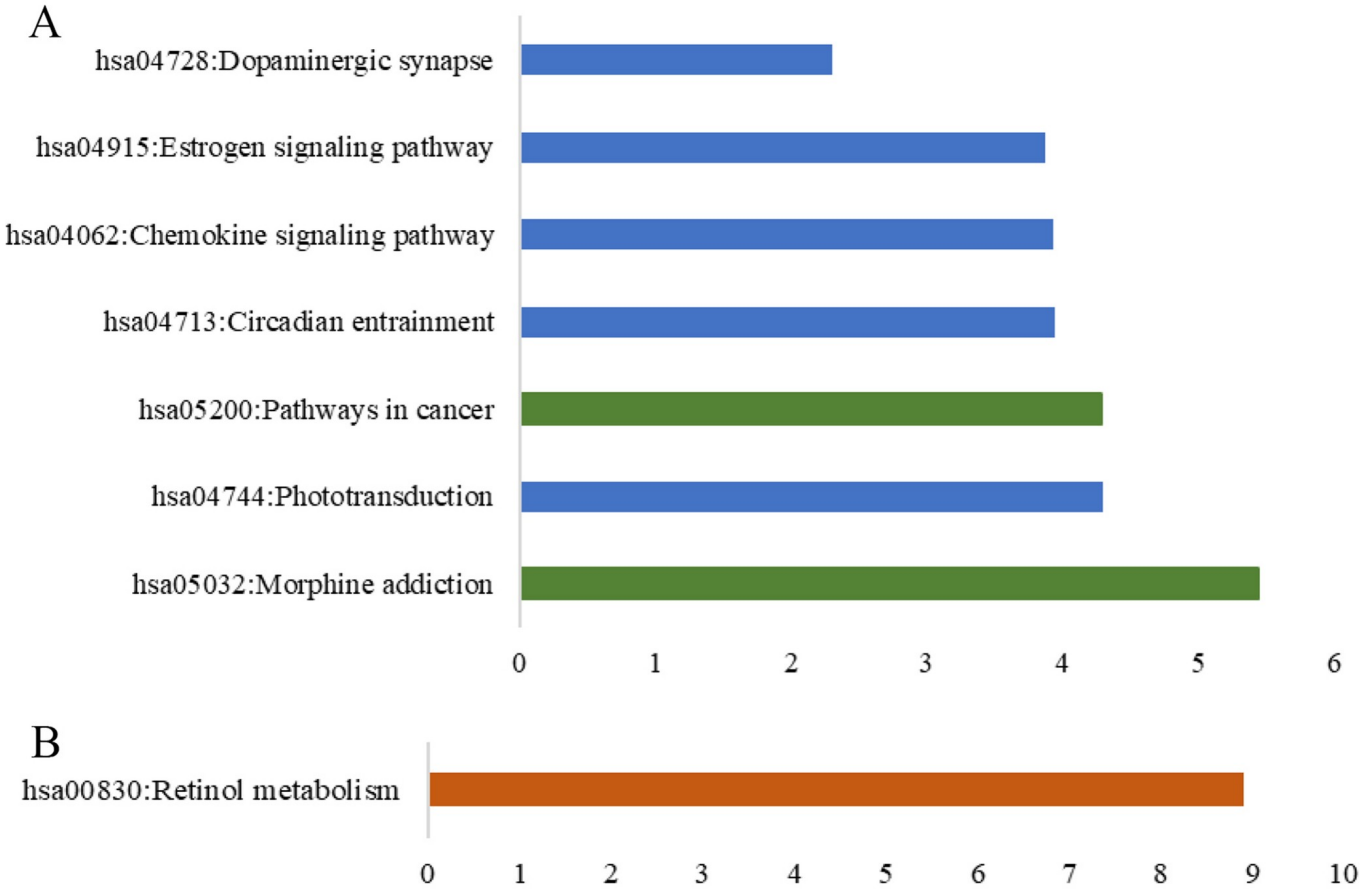
Enriched KEGG pathways. A. Signaling pathways involved in *GRK4* and its interacting proteins. Blue represents Organismal Systems, and green represents Human Diseases; B. Signal pathways involved in *RDH8* and its interacting proteins. Red represents Metabolism.

In order to explore potential effects of GRK4 and RDH8 on hypertension pathogenesis, a PPI network was constructed. Firstly, proteins potentially interacting with GRK4 or RDH8 were mined through 7 protein-interaction databases (Fig 3). Totally, 24 candidates might interact with GRK4; whereas 11 candidates with RDH8. Among them, 5 candidates interacting with GRK4 were associated with hypertension. Secondly, pathways were explored to predict how interaction proteins participate in signal transduction. Interestingly, GRK4 interacting proteins yielded 7 pathways (p <0.01), of which 5 pathways related to Organismal Systems and 2 pathways related to Human Diseases. RDH8 interacting proteins yielded one pathway related to Metabolism (Fig 4). GRK4 and RDH8 may interact with these proteins involved in hypertension. For example, based on enrichment analysis of *RDH8* interaction genes, *LRAT*, *AOX1*, *CYP26A1*, *BCO1*, *PNPLA4*, and *RETSAT* were involved in retinol metabolism. Downregulation of *LRAT* and a low level of serum retinol (VA) were independent predictors of EH. *LRAT* may affect blood pressure by down-regulating Ang II [29].

## Discussion

Hypertension is attributed to multiple environmental (such as obesity and excessive drinking) and genetic factors [30, 31]. Accumulating evidence indicates that genetic risk factors for hypertension vary with ethnicity and regions. For example, *CAMK4* rs10491334 is associated with hypertension in Uyghur population; whereas *GRK4* rs180105 in East Asians and Europeans; and *GRK4* rs2960306 in Europeans [32]. Abnormalities in SNS and RAS functions contribute to pathogenesis of hypertension. Therefore, among Han population in Xinjiang, SNS, RAS and candidate genes are hypothesized to play roles in etiology of hypertension.

In order to verify this conjecture, we have collected 151 hypertension cases and 65 controls, selected 54 genes related to SNS and RAS for targeted sequencing, and identified SNPs of genes related to hypertension. Notably, *GRK4* rs1801058 (*p*.*V486A*) and *RDH8* rs1644731 (*p*.*M222T*) were related to hypertension. Upon mutation was introduced, 3D protein structure, potential energy, RNA secondary structure, respectively, was evaluated. In order to explore functions of *GRK4* and *RDH8*, seven databases, such as String and HPRD, were used to mine proteins interacting with *GRK4* and *RDH8*, and to construct PPI network. Five proteins might interact with *GRK4*, such as *A2ML1*. Based on meta-analyses, *A2ML1* was related to hypertension [33]; *ADRB2* and *NOS3* jointly acted on sympathetic nervous system [34]; while *ADRB2* was related to hypertension in American Indians [35] and involved in pathophysiology of hypertension in Yi population [36]. In addition, transmembrane helix in *RDH8* might change to a certain extent due to *RDH8 p*.*M222A* mutation, which might affect protein transmembrane transport.

*GRK4* and *ADRB2* may play synergistic roles in hypertension through dysregulating SNS. Then, functional enrichment was analyzed by DAVID, and proteins interacting with *GRK4* were annotated to 7 KEGG pathways. Morphine Addiction process indirectly enhances the release of dopamine from dopaminergic synaptic terminals by relaxing inhibitory dopamine cells to discharge, while interacting proteins may affect the release of presynaptic dopamine terminals and enter postsynaptic cells in Dopaminergic Synapse. Dopamine receptor D1 is closely related to hypertension [37]. GRK4- interacting proteins may affect susceptibility to hypertension through morphine addiction and dopaminergic synaptic pathway.

RDH8 is involved in several GO terms such as retinol metabolism and NADP-retinol dehydrogenase catalysis. Retinol metabolism includes retinol chemical reactions and pathways. Retinol is required to produce vitamin A. NADP-retinol dehydrogenase catalyzes vitamin A binding to NADP^+^, and vitamin A is dehydrogenated into retinoids. In a recent GWAS study, pathways and biological processes related to hypertension were discovered, including retinoids metabolism [38], related to RDH8 and NADP-retinol dehydrogenase and thus vitamin A metabolism. NADP retinol dehydrogenase is a part of retinol metabolism, while retinol metabolism is a process of vitamin A metabolism. Low expression of LRAT, which may interact with RDH8, is an independent predictor of essential hypertension. RDH8 may participate in two biological processes, vitamin A metabolism and hypertension. Another interaction protein of *RDH8*, BCO1 (Beta-carotene 15,15’-dioxygenase), was related to lycopene [39], and thus hypertension [40].

In summary, *GRK4* and *RDH8* are genetic risk factors for essential hypertension in Han Chinese in Xinjiang. This finding provides new evidence for a better understanding of genetic factors susceptible for essential hypertension pathogenesis in Han Chinese in Xinjiang.

## Supporting information

S1 Fig Secondary structure of gene. A. secondary structure of GRK4 m.1457T; B. secondary structure of GRK4 m.1457C; C. secondary structure of RDH8 m.665T; D. secondary structure of GRK4 m.665C.
(DOCX)

S1 Table Targeted sequencing genes. (DOCX)

S2 Table Agilent company targeted sequence capture kit information. (DOCX)

S1 File SNV and Indel data of 151 patients and 65 healthy adults targeted capture sequencing. (RAR)
