# Supplementary material for: Targeted capture sequencing identifies genetic variations of GRK4 and RDH8 in Han Chinese with essential hypertension in Xinjiang
Source: PLoS One. 2021 Jul 23;16(7):e0255311. doi: 10.1371/journal.pone.0255311 (PMC8301621; doi:10.1371/journal.pone.0255311)
Supplement: S1 Fig — A. secondary structure of GRK4 m.1457T; B. secondary structure of GRK4 m.1457C; C. secondary structure of RDH8 m.665T; D. secondary structure of GRK4 m.665C. (DOCX) [file pone.0255311.s001.docx]

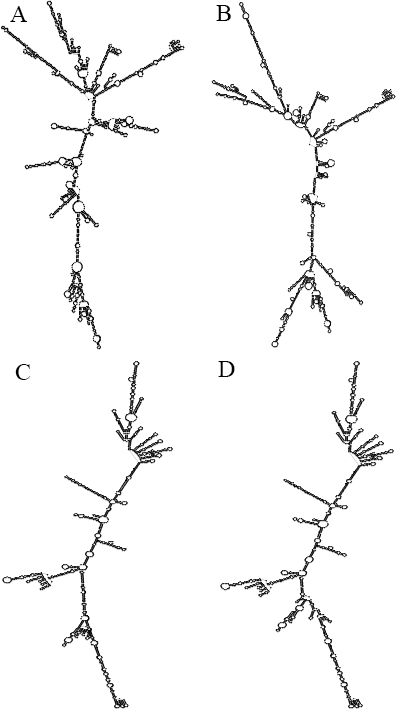


S1 Fig. A. secondary structure of GRK4 m.1457T; B. secondary structure of GRK4 m.1457C; C. secondary structure of RDH8 m.665T; D. secondary structure of GRK4 m.665C.
