# Supplementary material for: Targeted capture sequencing identifies genetic variations of GRK4 and RDH8 in Han Chinese with essential hypertension in Xinjiang
Source: PLoS One. 2021 Jul 23;16(7):e0255311. doi: 10.1371/journal.pone.0255311 (PMC8301621; doi:10.1371/journal.pone.0255311)
Supplement: S1 Table — (DOCX) [file pone.0255311.s002.docx]

S1 Table. Targeted sequencing genes

| Official Symbol | NCBI Gene | Chromosomal location | Reference sequence length (b) |
| --- | --- | --- | --- |
| NRF-1 | 4899 | 7q32 | 149380 |
| NRF-2 | 4780 | 2q31 | 38828 |
| TFAM | 7019 | 10q21 | 18087 |
| TFB1M | 51106 | 6q25.1-q25.3 | 71259 |
| TFB2M | 64216 | 1q44 | 29758 |
| TH | 7054 | 11p15.5 | 12208 |
| DDC | 1644 | 7p12.2 | 111021 |
| DBH | 1621 | 9q34 | 26981 |
| PNMT | 5409 | 17q12 | 6494 |
| COMT | 1312 | 22q11.21 | 32235 |
| MAO-A | 4128 | Xp11.3 | 95917 |
| ACE | 1636 | 17q23.3 | 25319 |
| ACE2 | 59272 | Xp22 | 111667 |
| ENPEP | 2028 | 4q25 | 91264 |
| CDKN2B-AS1 | 100048912 | 9p21.3 | 130306 |
| EMILIN1 | 11117 | 2p23.3-p23.2 | 11837 |
| ANPEP | 290 | 15q25-q26 | 33959 |
| REN | 5972 | 1q32 | 15521 |
| CMA1 | 1215 | 14q11.2 | 7344 |
| CTSG | 1511 | 14q11.2 | 6742 |
| CTSA | 5476 | 20q13.1 | 11868 |
| CPA3 | 1359 | 3q24 | 35831 |
| THOP1 | 7064 | 19p13.3 | 32093 |
| NLN | 57486 | 5q12.3 | 111087 |
| MME | 4311 | 3q25.2 | 163605 |
| AGT | 183 | 1q42.2 | 16067 |
| MAS1 | 4142 | 6q25.3-q26 | 5134 |
| AGTR2 | 186 | Xq22-q23 | 8267 |
| AGTR1 | 185 | 3q24 | 49132 |
| LNPEP | 4012 | 5q15 | 97769 |
| MTHFR | 4524 | 1p36.3 | 24373 |
| MTRR | 4552 | 5p15.31 | 53938 |
| Enos | 4846 | 7q36 | 27543 |
| ADRB1 | 153 | 10q25.3 | 6861 |
| ADRB2 | 154 | 5q31-q32 | 6041 |
| ADRB3 | 155 | 8p12 | 7671 |
| ADRA1A | 148 | 8p21.2 | 132161 |
| ADRA1B | 147 | 5q33.3 | 73445 |
| ADRA1D | 146 | 20p13 | 32381 |
| ADRA2A | 150 | 10q25.2 | 7875 |
| ADRA2B | 151 | 2q11.1 | 7370 |
| ADRA2C | 152 | 4p16 | 5957 |
| DRD1 | 1812 | 5q35.1 | 7488 |
| DRD2 | 1813 | 11q23 | 69684 |
| DRD3 | 1814 | 3q13.3 | 75610 |
| DRD4 | 1815 | 11p15.5 | 7401 |
| DRD5 | 1816 | 4p16.1 | 6375 |
| GNB3 | 2784 | 12p13 | 10541 |
| GNAS | 2778 | 20q13.3 | 75455 |
| GRK4 | 2868 | 4p16.3 | 81247 |
| MTR | 4548 | 1q43 | 113677 |
| DNMT1 | 1786 | 19p13.2 | 65734 |
| DNMT3a | 1788 | 2p23 | 113629 |
| DNMT3b | 1789 | 20q11.2 | 50971 |
