# Supplementary material for: Targeted capture sequencing identifies genetic variations of GRK4 and RDH8 in Han Chinese with essential hypertension in Xinjiang
Source: PLoS One. 2021 Jul 23;16(7):e0255311. doi: 10.1371/journal.pone.0255311 (PMC8301621; doi:10.1371/journal.pone.0255311)
Supplement: S2 Table — (DOCX) [file pone.0255311.s003.docx]

S2 Table. Agilent company targeted sequence capture kit information

| Target ID | Regions | Coverage | High Coverage  (≥90%) | Low Coverage  (<90%) |
| --- | --- | --- | --- | --- |
| chr10:111075032-111082907 | 1 | 75.71% | 0 | 1 |
| chr10:114042047-114048908 | 1 | 96.58% | 1 | 0 |
| chr10:58383134-58401230 | 1 | 67.50% | 0 | 1 |
| chr11:113407595-113477279 | 1 | 83.97% | 0 | 1 |
| chr11:2161929-2174137 | 1 | 100% | 1 | 0 |
| chr11:635305-642706 | 1 | 94.26% | 1 | 0 |
| chr12:6838854-6849395 | 1 | 62.61% | 0 | 1 |
| chr14:24503346-24510690 | 1 | 96.12% | 1 | 0 |
| chr14:24571518-24578260 | 1 | 82.49% | 0 | 1 |
| chr15:89782895-89816854 | 1 | 69.38% | 0 | 1 |
| chr17:39665981-39672475 | 1 | 98.68% | 1 | 0 |
| chr17:63475061-63500380 | 1 | 68.62% | 0 | 1 |
| chr19:10131345-10197079 | 1 | 60.93% | 0 | 1 |
| chr19:2783508-2815601 | 1 | 88.27% | 0 | 1 |
| chr1:11783730-11808103 | 1 | 82.30% | 0 | 1 |
| chr1:204152816-204168337 | 1 | 83.11% | 0 | 1 |
| chr1:230700523-230716590 | 1 | 71.81% | 0 | 1 |
| chr1:236792304-236905981 | 1 | 82.38% | 0 | 1 |
| chr1:246538561-246568319 | 1 | 84.23% | 0 | 1 |
| chr20:32760385-32811356 | 1 | 67.48% | 0 | 1 |
| chr20:4218631-4251012 | 1 | 86.60% | 0 | 1 |
| chr20:45888952-45900820 | 1 | 73.78% | 0 | 1 |
| chr20:58837740-58913195 | 1 | 88.97% | 0 | 1 |
| chr22:19939740-19971975 | 1 | 89.83% | 0 | 1 |
| chr2:177228303-177267131 | 1 | 86.50% | 0 | 1 |
| chr2:25230961-25344590 | 1 | 77.12% | 0 | 1 |
| chr2:27076567-27088404 | 1 | 83.67% | 0 | 1 |
| chr2:96110875-96118245 | 1 | 95.06% | 1 | 0 |
| chr3:114125797-114201407 | 1 | 96.78% | 1 | 0 |
| chr3:148695871-148745003 | 1 | 86.30% | 0 | 1 |
| chr3:148863256-148899087 | 1 | 95.84% | 1 | 0 |
| chr3:155022124-155185729 | 1 | 81.93% | 0 | 1 |
| chr4:110474073-110565337 | 1 | 64.24% | 0 | 1 |
| chr4:2961505-3042752 | 1 | 75.23% | 0 | 1 |
| chr4:3764569-3770526 | 1 | 95.00% | 1 | 0 |
| chr4:9779634-9786009 | 1 | 92.79% | 1 | 0 |
| chr5:148824593-148830634 | 1 | 84.59% | 0 | 1 |
| chr5:159914733-159988178 | 1 | 85.06% | 0 | 1 |
| chr5:175438672-175446160 | 1 | 59.41% | 0 | 1 |
| chr5:65720196-65831283 | 1 | 69.98% | 0 | 1 |
| chr5:7849186-7903124 | 1 | 95.81% | 1 | 0 |
| chr5:96933642-97031411 | 1 | 68.80% | 0 | 1 |
| chr6:155245238-155316497 | 1 | 71.09% | 0 | 1 |
| chr6:159904942-159910076 | 1 | 63.06% | 0 | 1 |
| chr7:129609702-129759082 | 1 | 60.63% | 0 | 1 |
| chr7:150989056-151016599 | 1 | 72.24% | 0 | 1 |
| chr7:50456436-50567457 | 1 | 90.17% | 1 | 0 |
| chr8:26740847-26873008 | 1 | 69.28% | 0 | 1 |
| chr8:37960995-37968666 | 1 | 80.45% | 0 | 1 |
| chr9:133634363-133661344 | 1 | 81.46% | 0 | 1 |
| chr9:21992791-22123097 | 1 | 81.79% | 0 | 1 |
| chrx:116168705-116176972 | 1 | 77.07% | 0 | 1 |
| chrx:15492402-15604069 | 1 | 90.32% | 1 | 0 |
| chrx:43652907-43748824 | 1 | 89.57% | 0 | 1 |
